# Broad Antibody Mediated Cross-Neutralization and Preclinical Immunogenicity of New Codon-Optimized HIV-1 Clade CRF02_AG and G Primary Isolates

**DOI:** 10.1371/journal.pone.0023233

**Published:** 2011-08-04

**Authors:** Simon M. Agwale, Joseph C. Forbi, Frank Notka, Terri Wrin, Jens Wild, Ralf Wagner, Hans Wolf

**Affiliations:** 1 Clinical Virology Laboratory, Innovative Biotech, Keffi/Abuja, Nigeria; 2 Frederick Innovative Technology Center, Innovative Biotech, Frederick, Maryland, United States of America; 3 GeneArt AG, Regensburg, Germany; 4 Monogram Biosciences Inc., South San Francisco, California, United States of America; 5 Molecular Microbiology and Gene Therapy Unit, Institute for Medical Microbiology and Hygiene, University of Regensburg, Regensburg, Germany; George Mason University, United States of America

## Abstract

Creation of an effective vaccine for HIV has been an elusive goal of the scientific community for almost 30 years. Neutralizing antibodies are assumed to be pivotal to the success of a prophylactic vaccine but previous attempts to make an immunogen capable of generating neutralizing antibodies to primary “street strain” isolates have resulted in responses of very limited breadth and potency. The objective of the study was to determine the breadth and strength of neutralizing antibodies against autologous and heterologous primary isolates in a cohort of HIV-1 infected Nigerians and to characterize envelopes from subjects with particularly broad or strong immune responses for possible use as vaccine candidates in regions predominated by HIV-1 CRF02_AG and G subtypes. Envelope vectors from a panel of primary Nigerian isolates were constructed and tested with plasma/sera from the same cohort using the PhenoSense HIV neutralizing antibody assay (Monogram Biosciences Inc, USA) to assess the breadth and potency of neutralizing antibodies. The immediate goal of this study was realized by the recognition of three broadly cross-neutralizing sera: (NG2-clade CRF02_AG, NG3-clade CRF02_AG and NG9- clade G). Based on these findings, envelope gp140 sequences from NG2 and NG9, complemented with a gag sequence (Clade G) and consensus tat (CRF02_AG and G) antigens have been codon-optimized, synthesized, cloned and evaluated in BALB/c mice. The intramuscular administration of these plasmid DNA constructs, followed by two booster DNA immunizations, induced substantial specific humoral response against all constructs and strong cellular responses against the gag and tat constructs. These preclinical findings provide a framework for the design of candidate vaccine for use in regions where the HIV-1 epidemic is driven by clades CRF02_AG and G.

## Introduction

Globally, approximately 33.4 million individuals now live with human immunodeficiency virus type-1 (HIV-1) infection, 22 million of whom reside in Sub-Saharan Africa. Thus, Sub-Saharan Africa remains the region most heavily affected by HIV. In 2008, sub-Saharan Africa accounted for 67% of HIV infections worldwide, 68% of new HIV infections among adults and 91% of new HIV infections among children. The region also accounted for 72% of the world's AIDS-related deaths in 2008 [1]. The development of a vaccine to prevent HIV infection is a global health priority. Previous clinical efficacy trials failed to support the continued development of recombinant gp120 (rgp120) as a candidate HIV vaccine since they were unable to elicit consistent T-cell or protective antibody responses [2], [3], [4], [5], [6]. However, the partial efficacy shown by the recent RV144 trial, the RV144 largest HIV vaccine clinical trial to date, has rekindled interest in rgp120 subunit vaccines [7]. The AIDSVAX B/E rgp120 vaccine used in the RV144 trial in Thailand is considered inappropriate for clinical trials in sub-Sahara Africa where the genetic diversity of group M HIV-1 is highest and where the epidemic is driven by HIV-1 clades different from those found in Thailand as well as to a lesser extent HIV-2 [8].

HIV-1 subtypes A and C have accounted for the majority of infections in the central and Southern region of Africa, whereas HIV-1 subtypes A, G and O have predominated in the Western regions of Africa. Nigeria is also experiencing a unique HIV-1 epidemic consisting of two highly divergent subtypes: CRF02_AG and G [8], [9]. The CRF02_AG is also most prevalent in West Africa and Central West African countries [10], [11], [12], therefore, Nigeria's success in promoting efforts towards HIV/AIDS prevention, treatment and vaccine development will be important for the region as a whole. The ability of vaccine-induced antibodies to neutralize primary isolates is at least partially related to the genetic closeness of the primary virus to the immunizing strain and geographic preference of many HIV subtypes [13], [14]. Accordingly, in order to contend with the high HIV sequence variation, there is great interest in selecting region-specific immunogens to maximize the likelihood of protection against local strains in the geographical area where the vaccine is intended for use. This kind of approach is adequate to additionally consider co-receptor usage, neutralization susceptibility, or neutralization potency of the serum from the individual from whom the isolate was obtained.

With over 30 years of active research, an effective HIV-1 vaccine that can be used for prophylactic or therapeutic purposes in humans has not been identified. The introduction of highly active antiretroviral therapies (HAART) has significantly reduced HIV morbidity and mortality [15]. HAART therapies are only effective in delaying the onset of disease or progression to AIDS but cannot eradicate the virus and the epidemic. The most effective way of intervention against viral diseases is by vaccination. With this subtype scenario, HIV/AIDS vaccine constructs would have to be developed against indigenous subtypes. It is, therefore, likely that HIV-1 subtype surveillance will remain an important component of vaccine design; as viral epitopes evolve, novel epitopes will need to be considered for eliciting potent antiviral immune responses.

It has long been believed that neutralizing antibodies are key to the success of a prophylactic vaccine. However, the enormous variability together with the extraordinary capability of HIV to escape the immune system impeded the successful development of protective env-based immunogens and cross-neutralization is hardly observed and predominantly restricted to closely related isolates [16], [17] . This observation has resulted in the generation of region-specific immunogens to maximize the likelihood of protection against local strains. This study was carried out to characterize viruses from HIV-1^+^ patients in Nigeria, for the presence of broadly neutralizing antibodies against subtype regional isolates. A selection of envelop sequences derived from primary HIV isolates of the Nigerian cohort was used for neutralization studies and cross neutralization was evaluated within the cohort testing pseudo-typed reporter viruses against individual plasma/sera samples. Envelopes of CRF02_AG and G, which were capable of stimulating broadly neutralizing antibodies, were further characterized. The overall ambition towards developing a regional vaccine candidate prompted us to expand the set of test antigens in order to include epitopes derived from various viral gene products offering different immune stimulatory capacities, necessary to activate different arms of the immune system. Therefore, supplementing the env candidates, gag and consensus tat genes were included in the set of antigens, which were codon-optimized and synthesized for use in a DNA-immunization study in mice in order to determine their immunogenicity and suitability as possible future vaccine candidates.

## Materials and Methods

### Study population

Blood samples were randomly collected from 23 individuals enrolling in the Nigerian national antiretroviral therapy program (Table 1). Blood specimens were separated by centrifugation into plasma and peripheral blood mononuclear cells (PBMC) using BD Vacutainer® CPT™ Tubes (BD Company, NJ USA). Plasma and PBMC were stored at −80°C and the plasma were used to build viral env vectors and test for neutralizing antibodies according to established standard operating procedures in the CLIA/CAP certified Clinical Reference Laboratory (MCRL) at Monogram Biosciences, San Francisco, California. Samples were collected as part of the national HIV treatment program and signed informed consent was obtained from all subjects. The program was approved by the Nigerian federal ministry of Health.

**Table 1:** Viruses and plasmas used in antibody neutralization assays.

Sample Identifier	Tested in Neutralization Assay		gp160 Sequenced	NT activity
	Serum/Plasma	Viral env		
NG1	X			Weak
NG2	X	X	X	Broad/Strong
NG3	X		X	Broad/Strong
NG4	X			Limited/Weak
NG5	X			Limited/Weak
NG6	X	X		Limited/Weak
NG7	X			Limited/Weak
NG8	X			Limited/Weak
NG9	X	X	X	Broad/Strong
NG10	X			Limited/Weak
NG11	X	X		Limited/Weak
NG12	X			Limited/Weak
NG13	X	X	x	Limited/Weak
NG14	X			Limited/Weak
NG15	X			Limited/Weak
NG16	X	X		Limited/Weak
NG17	X	X		Limited/Weak
NG18	X	X	x	Limited/Weak
NG19	X	X	x	Limited/Weak
NG20	X			Limited/Weak
NG21	X			Limited/Weak
NG22	X	X		Limited/Weak
NG23	X	X		Limited/Weak

### Neutralization assay

**Figure 1:**
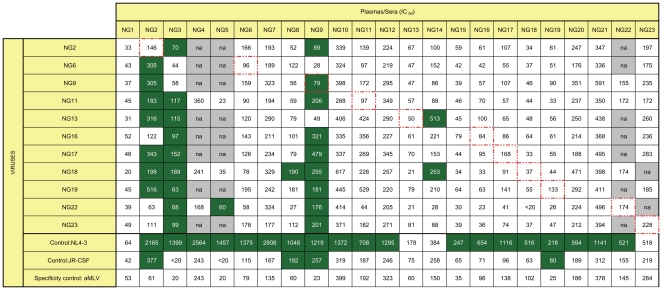
Neutralizing antibody titers of plasmas tested with autologous and heterologous viral envelopes. Plasma samples are from chronically infected individuals not currently on treatment. Titers are expressed as 1/plasma dilution. Higher numbers indicate greater inhibition (titer). Green color indicates positive neutralization activity which is significantly greater than inhibition of the specificity control, aMLV. Dashed red line indicates autologous plasma/virus pair. na = not available.

The PhenoSense™ HIV assay [18] initially developed to evaluate protease inhibitors and reverse transcriptase inhibitors was used in this study to measure virus-antibody neutralization. Briefly, HIV genomic RNA was isolated from virus stocks or plasma. DNA spanning the open reading frame of gp160 was amplified by reverse transcription PCR using forward and reverse primers located immediately upstream and downstream of the *env* initiation and termination codons, respectively. *env* PCR products were digested and ligated to compatible ends in an expression vector (pCXAS), expanded by transformation in competent *Escherichia coli* cells and plasmid DNA was purified from bacterial cultures. Virus particles containing patient virus Env proteins were produced by co-transfecting HEK293 cells (HEK293 cells: received from ATCC (commercial source: LGC Standards) (ATCC® Number: CRL-1573T, reference: [19], PubMed: 886304) with *env* libraries plus an *env^−^* HIV genomic vector that contains a firefly luciferase indicator gene. The HIV-1 genomic vector is replication defective and contains a luciferase expression cassette within a deleted region of the HIV envelope gene. Recombinant viruses pseudotyped with patient-derived virus Env proteins were harvested 48 h post-transfection and incubated for 1 h at 37°C with serial fourfold dilution of heat-inactivated patient plasma samples. U87 cells (U87: received from ATCC (commercial source: LGC Standards) (ATCC® Number: HTB 14T, reference: [20] , PubMed: 4313504) that express CD4 plus the CCR5 and CXCR4 co-receptors were inoculated with virus-plasma dilutions. Virus infectivity was determined 72 h post inoculation by measuring the amount of luciferase activity expressed in infected cells by comparing the amount of luciferase activity produced in the presence of test serum/plasma to the amount of luciferase activity produced in absence of test serum/plasma. Neutralizing activity was displayed as the percent inhibition of viral replication (luciferase activity) at each antibody dilution compared to an antibody negative control [21]: % inhibition = {1−[(luciferase with antibody/luciferase without antibody)}×100. Titers were calculated as the reciprocal of the plasma dilution conferring 50% inhibition (IC_50_) (Figure 1).

### Test Viruses and Plasmas

A panel of recombinant viruses was prepared from patient plasma collected from Nigeria and where classified as Group M, subtype CRF02_AG and G [18], [21]. The same sera/plasma used to isolate virus was also used for neutralization tests with the autologous isolate as well as with heterologous isolates from the same study group. Additionally, control viruses NL4-3, JR-CSF and amphotropic Murine Leukemia Virus envelope (aMLV) were tested with the sera. NL4-3 is a T-cell line adapted isolate that is extremely neutralization-sensitive and is used as a control to detect the presence of even low amounts of neutralizing antibody in samples. JR-CSF is a minimally passaged R5 tropic virus, and is less sensitive to neutralization. It is more representative of primary field isolates than the T-cell line adapted viruses while aMLV is the specificity control and is tested with every plasma/serum. This control functions to indicate the presence of non-HIV specific factors, such as toxicity or serum proteins that may influence infectivity of the viruses. aMLV env proteins are able to mediate virus entry in U87/CD4/CCR5/CXCR4 cells but are not inhibited by anti-HIV Env antibodies. Scoring of a positive reaction requires that the IC_50_ for a serum/plasma be at least 3-fold higher than the IC_50_ of the same serum with aMLV. Patient plasma evaluation was started at a dilution of 1∶10 in cell culture medium followed by 10 serial 4-fold dilutions. An equal volume of recombinant virus stock was added to the diluted plasma/serum making the final neutralization reaction concentrations as follows: 1∶20, 1∶80, 1∶320, 1∶1.280, 1∶5.120, 1∶20.480, 1∶81.920, 1∶327.680, 1∶1.310.720, 1∶5.242.880. U87 cells expressing CD4/CCR5/CXCR4 or a mixture of cells expressing CD4/CCR5 and CD4/CXCR4 were added to the reaction following a 1-h incubation of the serum dilutions and virus at 37°C. Infected cells were incubated for 3 days and luciferase expression was evaluated on the final day.

### Construction of plasmid DNA immunization constructs

In order to evaluate the immunogenicity of isolates from individuals who had developed broadly neutralizing activity, gp140 of two viruses, one belonging to subtype CRF02_AG and the other belonging to the G-subtype were synthesized, cloned and evaluated preclinically in BALB/c mice. The ‘Cobra’ vector pORT1 was selected for DNA delivery because it does not contain an antibiotic resistance gene making it a candidate vector for planned future clinical trials. The genes were optimised for mammalian expression, synthetically generated and sub-cloned into the pORT1 plasmid. The details of this method have been described previously [22]. Furthermore, four different DNA-vaccine prototype constructs based on HIV-1 clade CRF02_AG and G reading frames (ConcTat NG, gag 203, env NG2 and env NG9) were evaluated. The gag 203 sequence was derived from an HIV-1 subtype G infected Nigerian patient while the consensus tat NG was assembled from sequences derived from seven Nigerian patients infected with either HIV-1 clade CRF02_AG or G.

### Vaccination of mice

Female BALB/c mice (Charles River, Sulzfeld, Germany) were divided into six groups; each group comprised six animals. At the age of 8–12 week the BALB/c mice were vaccinated with 100 ug of each plasmid {pORT ConcTat NG, pORT Gag 203, pORT Env NG2, pORT Env NG9, pORT gp120 C (positive control), pORT1a clade (negative control) without adjuvant by intramuscular (i.m.) saline injection of 50 µl each in both site tibialis anterior muscles, followed by two booster vaccination with the same doses (4 and 7 weeks for first and second booster, respectively). At the indicated time points, individually earmarked mice were bled and at last sacrificed. Spleens of three mice were pooled and tested. Immunological parameters analysed were i) specific antibody generation as determined by western blotting for all four inserts and ii) activation of antigen specific T cells as determined by intracellular INFγ-staining of CD8^+^ T cells using FACS and quantification of INFγ-producing cells using ELISPOT.

All experiments on BALB/c mice were conducted in accordance with the legal requirements of local and national authorities (ethics review by government (via Ethics Committee) of Lower Franconia, Germany).

### Transfection of cells

Human lung carcinoma (NCI-H1299 NCI-H1299, received from ATCC (commercial source: LGC Standards) (ATCC® Number: CRL-5803T, reference: [23] PubMed: 1563005) cells were maintained in Dulbecco's modified Eagle medium (Invitrogen) supplemented with 10% fetal calf serum, 2 mM L-glutamine, 100 IU of penicillin, and 100 mg of streptomycin per milliliter (PAN Biotech, Aidenbach, Germany) and transfected (using calcium co-precipitation technique) as previously described [24]. Briefly, 3×10^6^ NCI-H1299 cells were seeded on 100 mm-diameter culture dishes, incubated for 24 h and then transfected with 45 µg of different Nucleobond AX (Macherey-Nagel, Düren, Germany) purified plasmid constructs. At 16 h post transfection, the cell culture supernatant was replaced with fresh medium. Cells were harvested 48 h after transfection.

### Immunoblotting

Total cell lysates were prepared 48 h post transfection using a triple detergent puffer system (RIPA) which was supplemented with a cocktail of protease inhibitors (Boehringer CompleteTM Mini Kit; Mannheim GmbH, Mannheim, Germany). Soluble protein was prepared and protein concentration was determined by Bradford assay. 75 µg of proteins were separated by SDS-PAGE and transferred to nitro-cellulose membranes. Blots were probed with different concentrations of pooled antisera derived from vaccinated mice (pre-immune and immune sera). An anti-mouse IgG AP-conjugated monoclonal antibody was used to detect specific proteins as described [25].

### Intracellular IFNγ staining and FACS analysis

IFNγ expression of CD8^+^ cells was detected by intracellular staining followed by FACS analysis. Spleenocytes were stimulated with 10 µM peptide pools (Mimotopes, Pty Ltd., Australia). The peptides were reconstituted with 13 µl DMSO resulting in an average concentration of 125 µg/µl. The pools were constructed by combining 2 µl of each peptide in RPMI medium. Medium alone served as the negative control. The peptides were incubated for 6 h with the cells in medium with Brefeldin A (5 mg/ml) for the whole incubation time. Intracellular staining was performed as described [26]. In total, 3×10^4^ CD8^+^ lymphocytes were analyzed by flow cytometry using a FACS Calibur and CellQuest software (BD Biosciences, San Jose, CA).

### ELISPOT assay

96-well multiscreen MAHA-S45 plates (Millipore, Eschborn, Germany) were coated with 50 µl of anti-mouse IFNγ ab (cat. no. 554431, BD, Heidelberg, Germany; 1∶500 PBS without Mg^++^/Ca^++^). ELISPOT assays (for IFNγ) were performed as previously described [26]. The plates were then air dried and colored spots were counted.

## Results

### Neutralization Results

The IC_50_s from neutralization reactions of 23 sera samples are shown in Figure 1. All sera except one (NG9 with low neutralizing titres; IC50 = 79), were negative against the homologous virus from the same patient blood samples. Seventeen sera were also negative when tested against the heterologous viruses in the panel. Nineteen patient samples, however, were positive with NL4-3, a virus which is very sensitive to the presence of neutralizing antibodies, indicating that even though the sera may have failed to cross-neutralize the primary isolates, neutralizing antibodies were present, as shown by inhibition of the T cell line adapted virus. Three patient plasma/sera samples showed extensive cross-neutralization of other primary isolates. These samples are NG2 (n = 11), NG3 (n = 12) and NG9 (n = 15). Although these three plasma/sera effectively neutralize NL4-3, we do not see a correlation between magnitude of IC_50_ against NL4-3 and cross-neutralization of other isolates. In many cases neutralization titers against NL4-3 were as high or higher in sera without broad cross-neutralization activity as in sera with broad cross reactivity. Several sera exhibited high background activity as shown by IC_50_s of >100 against aMLV (Figure 1). Since only a subset of the samples showed this effect; a general effect associated with cell toxicity is most probably not the cause of the non-specific inhibitor. A conceivable reason for the observed effect is the presence of unknown agents in the plasma samples causing inhibition of the virus through some unclear mechanisms, notably affecting NG7, NG10, NG12, NG64, NG21 and NG23. although the study subjects were assumed to not be on treatment at the time of the acquisition of the plasma, the presence of the high level background against all isolates including aMLV suggest that some may have been. gp160 nucleotide sequences of envelope genes of isolated virions from each 3 individuals with broad cross-neutralizing activity and 3 individuals with very low neutralizing antibody activity were sequenced (data not shown). Two individuals with broad cross-neutralizing antibodies were infected with CRF02_AG and one of and G, respectively. Two of the envelope genes (env NG2 and env NG9) derived from patients showing broad neutralizing activities belonging to HIV-1 clades CRF02_AG and one of the individual was infected with a subtype G virus, were chosen for further analyses using the BALB/c mouse model in order to evaluate their immunogenicity. To monitor the amount of neutralising activity that is not mediated by antibodies directed against HIV-1 env proteins, gag (clade G) and consensus tat (CRF02_AG and G) were also tested for their ability to generate humoral and cellular immunity in the same animal model.

### Humoral immune responses after the second and third immunization in mice

Regarding the humoral immune responses, HIV specific antibodies were found by western blot after the second immunisation in animals that received the ConcTat NG and gag 203 and after the third immunisation for all constructs (ConcTat NG, gag 203, env NG2 and env NG9). The western blots were performed using lysates of cells transiently transfected with the corresponding pORT1-antigen expression constructs. No antibodies were detected in the negative control mice (pORT1a clade). In contrast, the positive control (pORT gp120 C) induced substantial specific antibodies.

### Cellular immune responses in mice

**Figure 2:**
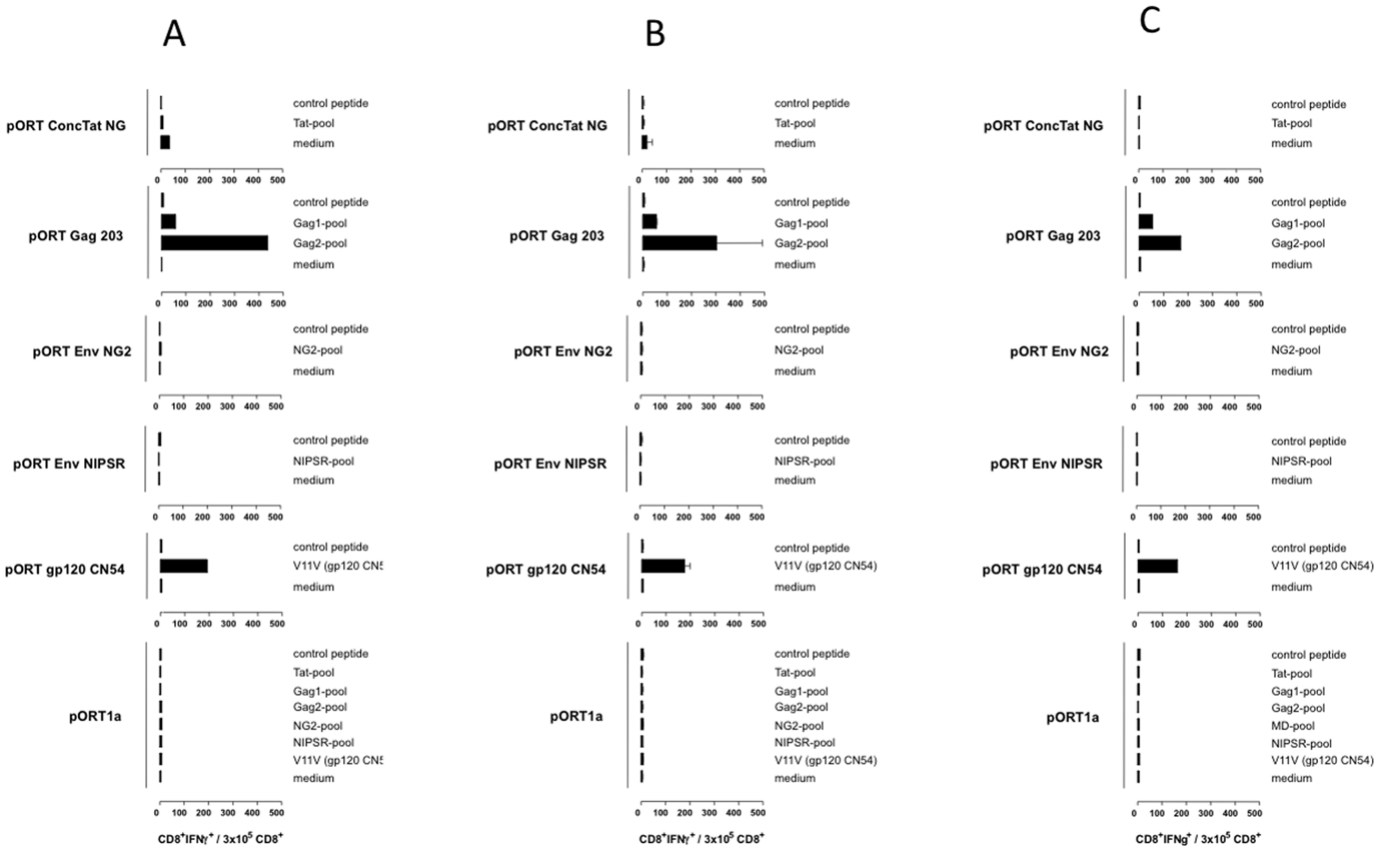
Cellular immune responses induced by the DNA vaccine candidates. Intra-cellular INF gamma (IFNγ) staining IFNγ production was measured by FACS analysis after intracellular staining of IFNγ). Balb/C mice (n = 6 per group) were inoculated IM with (i) pORT ConcTat NG, (ii) pORT Gag 203 , (iii) pORT Env NG2 , (iv) pORT Env NG9 , (v) pORT gp120 C N54 (positive control), (vi) pORT1a clade (negative control). One week after the second/third booster immunization, spleen cells were isolated and tested for specific cellular immune responses by measuring IFN-g production after stimulation with pools of overlapping 15-mer peptides. Shown is the total number of CD8^+^ IFNγ^+^ cells per 30.000 CD8^+^ restimulated mouse spleen cells. A) mice 1–3, B) mice 4–6, C) merged data.

**Figure 3:**
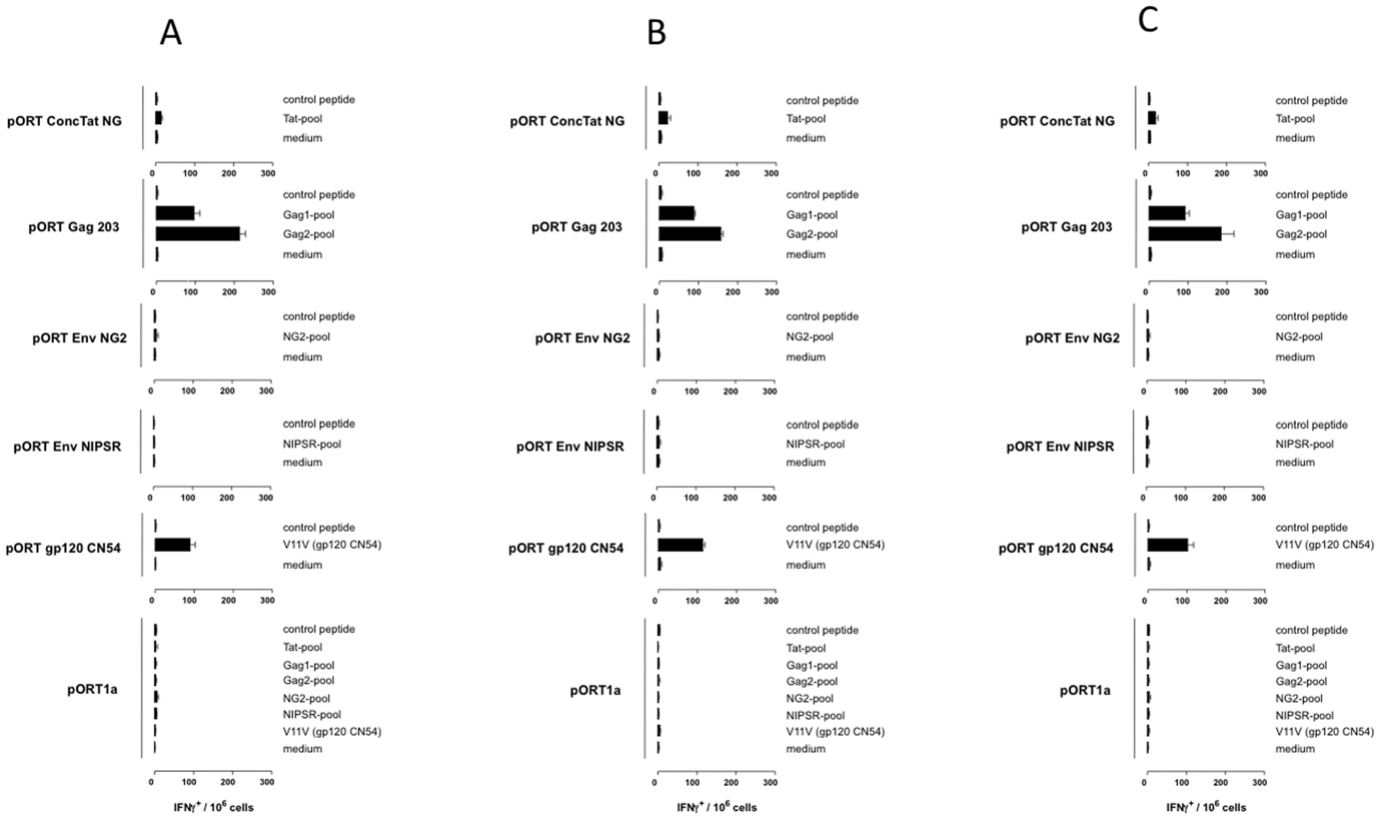
Cellular immune responses induced by the DNA vaccine candidates (IFNγ production was measured using a commercial ELISPOT assay). Balb/C mice (n = 6 per group) were inoculated IM with (i) pORT ConcTat NG, (ii) pORT Gag 203 , (iii) pORT Env MD , (iv) pORT Env NG9 , (v) pORT gp120 CN54 (positive control), (vi) pORT1a clade (negative control). One week after the second/third booster immunization, spleen cells were isolated and tested for specific cellular immune responses by measuring IFN-g production after stimulation with pools of overlapping 15-mer peptides. Shown is the total number of positive (IFNγ secreting) cells per 10^6^ restimulated mouse spleen cells. A) mice 1–3, B) mice 4–6, C) merged data.

Regarding the cellular immunity, the FACS and ELISPOT assays demonstrated the presence of very solid Gag-specific CD8^+^ T cell responses in mice immunized with pORT Gag 203. This was confirmed independently by the FACS analysis (Figure 2) and ELISPOT assay (Figure 3). For these assays mouse spleen cells were restimulated with overlapping peptides covering the complete gag 203 gene and separated into two pools, Gag1 and Gag2. Each of the pool induced positive responses, pool 2 higher than pool 1, arguing for either at least two CTL epitopes being present in Gag or one epitope localised at the “border” of the two pools. Similarly, for the tat gene a set of overlapping peptides covering the complete sequence (as contained in pORT ConcTat NG) was used for immunological evaluation assays, revealing only weak to moderate cellular immunity. The data can be interpreted firstly as no CTL epitope present (negative FACS data) and secondly as a very weak T helper response detectable (low ELISpot results). For the env constructs (pORT Env NG2 and pORT Env NG9) no cellular immune response could be detected. As expected the positive control (pORT gp120 C) showed a strong cellular immune response while no cellular immune response could be found in the negative control (pORT1a clade) (Figures 2 and 3).

## Discussion

Nigeria, with a population of over 140 million people, has the largest HIV/AIDS epidemic in Africa which is characterized by two HIV-1 subtypes, CRF02_AG and G [8], [9], [27]. It has long been recognized that, although HAART has been shown to be clinically effective, it does not rid the host of the virus, may be toxic and does not allow the reconstitution of potentially useful anti-HIV immune responses in infected patients [28]. A major goal in HIV-related research remains the identification of a potent prophylactic vaccine to prevent HIV infection. In this study, neutralizing antibodies against autologous and heterologous primary HIV-1 isolates were measured and found to be generally of low titer. This is likely due to the inability of the immune system in these individuals to efficiently respond to rapidly evolving quasi-species. Only patient NG9 showed autologous antibody response probably because cell-derived virus does not accurately reflect the actively replicating population present in plasma. Detection of autologous responses have been difficult to measure because of the technical challenges associated with the preparation of autologous virus stocks that are typically obtained from PBMCs [21].

Although neutralizing activity against autologous virus was generally low, neutralizing activity against isolates from other members of the study subjects (heterologous virus) was comparatively higher particularly NL4-3 (Figure 1). NL4-3 is a laboratory adapted strain that has presumably lost those virologic factors that protect against antibody-mediated neutralization *in vivo* [29]. NL4-3-specific responses may therefore be a surrogate for the overall level of HIV-specific neutralizing titers. Despite the generally low level of neutralizing antibody titers, three plasma did demonstrate a wide breath of solid cross neutralizing activity against a range of primary Nigerian viruses with high cross-neutralizing titers as shown (Figure 1), suggesting that these antibodies may be protective and could contribute to an effective HIV vaccine. This corroborates with previous studies showing that certain anti-V3 NAbs can neutralize diverse strains of HIV-1 [30], [31], [32]. The ability to cross-neutralize viruses isolated from other individuals as seen in sera from this study possibly implies that certain individuals do have neutralizing capabilities that could be taken advantage of in term of viral evolution and for vaccine design in regions with HIV-1 CRF02_AG and G epidemic. In addition, the individuals with broad antibody responses are usually chronically infected and the antibody breath results from either the ongoing production of HIV-specific antibodies in response to viral evolution or the maturation of the antibody response over time. Although the factors that contribute to the development of broad heterologous responses remain unclear, it has been speculated that broadly reactive responses evolve over a longer course of HIV infection [21], [33], [34]. Whether this observation reflects antigen-driven expansion and/or abnormal B-cell activation is unclear. The immediate goal of this study was realized by finding of three cross-neutralizing sera and the cloning of viral envelopes from those sera.

Within the presented study three cross-neutralizing sera have been identified and the respective virus isolates have been subjected to continuative preclinical analyses. Envelope gp140 sequences from the three isolates that generated these antisera have been codon-optimized and two (subtypes CRF02_Ag and G) were synthesized and cloned for preclinical evaluation in mice. We believe that this small animal model will provide a rapid and inexpensive means to improve the safety and effectiveness of proposed DNA vaccines. Furthermore, mice models have been shown in this study and others to elicit strong humoral and cell mediated responses against HIV [22], [35]. It is anticipated that these pre-clinical studies will provide additional experimental support to the HIV-1 subtypes CRF02_Ag and G vaccine endeavor and open the way to HIV phase 1 studies of a regional vaccine.

There is general consensus in the HIV vaccine field that successful prophylactic HIV-1 vaccine candidates will need to induce potent broad neutralizing antibodies (Nab) as well as cytotoxic T lymphocytes (CTL) [17]. Since DNA vaccines are comparatively faster and cheaper to develop and are not dependent on continuous cold-chain disposition for distribution, they provide a promising avenue for the development of new vaccination strategies. The technological principle of DNA vaccines relies on a cloned nucleic acid to encode an antigen that when taken up is expressed by the host cells in amounts sufficient to elicit an immune response strong enough to protect against infectious diseases. Such DNA vaccine plasmids can include multiple antigens/genes and recombinant DNA technology offers an easy way to mix-and-match antigens to produce novel combinations to stimulate an optimized response [22], [36], [37], [38].

We assessed the immunogenicity of a panel of primary HIV-1 isolates in BALB/c mice which included, pORT ConcTat NG (consensus tat clade CRF02_AG and G), pORT Gag 203 (clade G), pORT Env NG2 (clade G) and pORT Env NG9 (clade CRF02_AG). The humoral immune response was evaluated based on sera harvested one week after the first and second booster immunization. It is encouraging and interesting that all constructs elicited HIV specific antibody responses which is one major requirement for HIV vaccine. This was substantiated by the lack of detectable antibodies in the negative control mice. Using both the FACS and ELISPOT analyses robust Gag-specific CD8^+^ T cell responses were observed (Figures 2 and 3). Interestingly, the breath and magnitude of the cellular immune response detected for pORT Gag 203 was stronger than that observed for the positive control. Noteworthy was the Gag-specific cellular immune response restimulated by pool 2 which was stronger than that by pool 1 arguing for either response to at least two CTL- epitopes being present in Gag or for one epitope localised at the “border” of the two pools. This observation concurs with previous studies showing that Gag represents a major target of CTL responses in HIV-1 infected patients and has a high density of epitopes [39]. This observation gives impetus to vaccine design strategies that seek to elicit responses to a broad array of HIV-1 epitopes, and suggest a particular focus on Gag [40]. Also, the consensus Tat NG generated only moderate cellular immunity. It is commonly accepted that the use of combinations of HIV proteins as vaccine immunogens is more effective than a single antigen [22]. Given this, the combination of a Gag and Tat based vaccine may be reasonable. For the Env constructs (pORT Env NG2 and pORT Env NG9) no cellular immune response could be detected (Figures 2 and 3). However, the peptides used to detect cellular immunity represent only a small fragment of the Env V3 region and this experiment was restricted to a BALB/c mouse background. Therefore, additional experiments to evaluate the cellular immune responses against the Env constructs are planned. Collectively, this study demonstrates that a CRF02_AG and G based vaccine can stimulate humoral and cell mediated responses and should be explored further for possible use as a candidate vaccines. This provides a framework for the rational design of vaccines against HIV-1 in regions of West and West-central Africa.
